# Apalutamide efficacy, safety and wellbeing in older patients with advanced prostate cancer from Phase 3 randomised clinical studies TITAN and SPARTAN

**DOI:** 10.1038/s41416-023-02492-8

**Published:** 2023-11-11

**Authors:** John Shen, Simon Chowdhury, Neeraj Agarwal, Lawrence I. Karsh, Stéphane Oudard, Benjamin A. Gartrell, Susan Feyerabend, Fred Saad, Christopher M. Pieczonka, Kim N. Chi, Sabine D. Brookman-May, Brendan Rooney, Amitabha Bhaumik, Sharon A. McCarthy, Katherine B. Bevans, Suneel D. Mundle, Eric J. Small, Matthew R. Smith, Julie N. Graff

**Affiliations:** 1 Jonsson Comprehensive Cancer Center, University of California Los Angeles, Los Angeles, CA USA; 2 Guy’s, King’s, and St. Thomas’ Hospitals, and Sarah Cannon Research Institute, London, UK; 3 Huntsman Cancer Institute, University of Utah, Salt Lake City, UT USA; 4 The Urology Center of Colorado, Denver, CO USA; 5 Georges Pompidou Hospital, University of Paris Cité, Paris, France; 6 Montefiore Medical Center, Bronx, NY USA; 7 Studienpraxis Urologie, Nürtingen, Germany; 8 Centre Hospitalier de l’Université de Montréal, Université de Montréal, Montréal, QC Canada; 9 Associated Medical Professionals of NY, Syracuse, NY USA; 10 BC Cancer and Vancouver Prostate Centre, Vancouver, BC Canada; 11 Ludwig-Maximilians-University (LMU), Munich, Germany; 12 Janssen Research & Development, Spring House, PA USA; 13 Janssen Research & Development, High Wycombe, UK; 14 Janssen Research & Development, Titusville, NJ USA; 15 Janssen Research & Development, Raritan, NJ USA; 16 Janssen Research & Development, Horsham, PA USA; 17 Helen Diller Family Comprehensive Cancer Center, University of California San Francisco, San Francisco, CA USA; 18 Massachusetts General Hospital Cancer Center and Harvard Medical School, Boston, MA USA; 19 VA Portland Health Care System, Portland, and Knight Cancer Institute, Oregon Health & Science University, Portland, OR USA

**Keywords:** Prostate cancer, Outcomes research

## Abstract

**Background:**

Apalutamide plus androgen-deprivation therapy (ADT) improved outcomes in metastatic castration-sensitive prostate cancer (mCSPC) and non-metastatic castration-resistant PC (nmCRPC) in the Phase 3 randomised TITAN and SPARTAN studies, respectively, and maintained health-related quality of life (HRQoL). Apalutamide treatment effect by patient age requires assessment.

**Methods:**

Post-hoc analysis assessed patients receiving 240 mg/day apalutamide (525 TITAN and 806 SPARTAN) or placebo (527 TITAN and 401 SPARTAN) with ongoing ADT, stratified by age groups. Prostate-specific antigen declines, radiographic progression-free survival, metastasis-free survival, overall survival (OS), HRQoL and safety were assessed using descriptive statistics, Kaplan-Meier method, Cox proportional-hazards model and mixed-effects model for repeated measures.

**Results:**

Hazard ratios (95% confidence intervals) generally favoured apalutamide plus ADT versus ADT alone across all endpoints regardless of age; e.g., OS values were 0.57 (0.40–0.80), 0.70 (0.54–0.91) and 0.74 (0.40–1.39) (TITAN) and 0.39 (0.19–0.78), 0.89 (0.69–1.16) and 0.81 (0.58–1.15) (SPARTAN) in patients aged <65, 65–79 and ≥80 years. Regardless of age, apalutamide also maintained HRQoL and was tolerated well with a potential trend in rates of adverse events increasing with age. Limitations include post-hoc nature and variability in sample size of age groups.

**Conclusions:**

Apalutamide plus ADT was an effective and well-tolerated option maintaining HRQoL in patients with mCSPC and nmCRPC regardless of age.

**Clinical trial registration:**

TITAN (NCT02489318); SPARTAN (NCT01946204).

## Introduction

Advanced prostate cancer (PC), such as metastatic castration-sensitive PC (mCSPC) or non-metastatic castration-resistant PC (nmCRPC), generally affects older patients: the median age of patients with mCSPC and nmCRPC enrolled in clinical trials is approximately 68 and 74 years, respectively [1–7]; real-world populations are even older [8]. Treating older patients with PC is challenged by susceptibility to treatment complications and age-related comorbidities [9]. The International Society of Geriatric Oncology 2019 Guidelines recommend managing PC in older patients based on health status rather than on chronological age [10]. Hormonal interventions (e.g., androgen-deprivation therapy [ADT]) can increase risk of falls, osteoporotic fractures and development of frailty [11, 12], aggravating age-related conditions. Efficacy and safety of androgen receptor inhibitors and ADT combinations in older patients are being investigated [13, 14].

Guidelines highlight the importance of evaluating patient-reported outcomes and tolerability in older patients using well-defined and reliable instruments assessing health-related quality of life (HRQoL) [15, 16]. Functional Assessment of Cancer Therapy-Prostate (FACT-P) is a tool for assessing HRQoL in PC consisting of 39 items measuring physical, functional, emotional and social/family wellbeing, as well as concerns specific to PC [17].

A comprehensive assessment of apalutamide plus ADT in mCSPC and nmCRPC from the TITAN (NCT02489318) and SPARTAN (NCT01946204) placebo-controlled studies, respectively, showed that it improves clinical outcomes (e.g., long-term survival) [2, 7, 18, 19] and maintains HRQoL per FACT-P [20, 21]. Apalutamide treatment effect generally favoured point estimates of radiographic progression-free survival (rPFS) and overall survival (OS) in TITAN and metastasis-free survival (MFS) and OS in SPARTAN regardless of patients’ age, although age stratifications differed. In this post hoc analysis of TITAN and SPARTAN, we performed an in-depth assessment of apalutamide efficacy, tolerability, safety and patient-reported outcomes in advanced disease across uniformly stratified age groups.

## Methods

TITAN and SPARTAN were multicentre, Phase 3 randomised, double-blind, placebo-controlled studies of apalutamide plus ADT in mCSPC and nmCRPC, respectively, that randomised patients in 1:1 and 2:1 ratio to receive 240 mg/day apalutamide or placebo with concurrent ADT [2, 7]. TITAN was conducted at 260 sites in 23 countries; SPARTAN was conducted at 332 sites in 26 countries. Review boards at all participating institutions approved the studies, which were conducted according to current International Council for Harmonisation Guidelines for Good Clinical Practice and the principles of the Declaration of Helsinki. All patients provided written informed consent.

This post hoc analysis assessed apalutamide treatment effect on efficacy, HRQoL and safety, by patient age: <65, 65–79 and ≥80, or <75 and ≥75 years. Efficacy was assessed in the intent-to-treat (ITT) populations using rPFS and OS in TITAN, MFS and OS in SPARTAN, and best prostate-specific antigen (PSA) decline, defined according to Prostate Cancer Working Group 2 [22] criteria as a decline of ≥50% from baseline or decline to ≤0.2 ng/ml at any time during the studies and confirmed ≥4 weeks later. Patient-reported HRQoL was assessed in the ITT populations using FACT-P total score, FACT-P Physical Wellbeing subscale, and two items from Physical Wellbeing subscale GP1 “I have a lack of energy” and GP5 “I am bothered by side effects of treatment”. Response to FACT-P items were coded 0–4 (from “very much” to “quite a bit”, “sometimes”, “a little bit” and “not at all”); the sum for all responses ranges from 0 to 156, higher scores indicating better HRQoL [17, 23]. Changes from baseline of ≥10 points for FACT-P total,^24^ ≥ 3 points for FACT-P Physical Wellbeing [24], and ≥1 for GP1 or GP5 were considered clinically meaningful. GP1 and GP5 were considered independent of the FACT-P total score.

Treatment-emergent adverse events (TEAEs), defined as AEs occurring on or after first dose of the study drug through one cycle (30 days in TITAN and 28 days in SPARTAN) after the last study treatment, were assessed in the safety populations (patients who received the study drug). Additional details on study design are described in the Supplementary Methods.

### Statistical analysis

rPFS and MFS were analysed at first interim analysis cutoff that was prespecified to be final. OS, best PSA decline, HRQoL and safety were assessed at the final analysis cutoff that analysed crossover patients as a part of the ITT population in the placebo group. The sample size determination is described in Supplementary Methods.

Time-to-event endpoints were assessed using Kaplan–Meier methods and Cox proportional-hazards models. HRQoL was assessed as least squares mean changes from baseline in FACT-P total and item scores using a mixed-effects model for repeated measures at each scheduled visit during a treatment phase that had >10% of patients completing FACT-P. Baseline score, treatment, cycle and treatment-by-cycle interaction were fixed effects, individual patients were included as random effects, and missing data values were assumed to arise randomly. PSA decline, baseline characteristics, TEAEs and concomitant bone-sparing agent use were summarised descriptively.

## Results

### Patient populations

Between 15 December 2015 and 25 July 2017, 1052 patients were enrolled in TITAN, including 525 and 527 patients in apalutamide and placebo groups (Fig. S1a). The prespecified first interim analysis (final analysis for rPFS) occurred at the cutoff on 23 November 2018, after 22.7 months of median follow-up. The prespecified final analysis occurred at the cutoff on 7 September 2020, after the number of required deaths had occurred within 44.0 months of median follow-up.

Between 4 October 2013 and 15 December 2016, 1207 patients were enrolled in SPARTAN, including 806 and 401 patients in apalutamide and placebo groups (Fig. S1b). The prespecified final analysis for MFS occurred at the cutoff on 19 May 2017, after 20.3 months of median follow-up. The final analysis of OS occurred at the cutoff on 1 February 2020, after the number of required deaths had occurred within 52.0 months of median follow-up.

In TITAN, respectively, 331 (31%), 628 (60%) and 93 (9.0%) patients were aged <65, 65–79 and ≥80 years, and 806 (77%) and 246 (23%) patients were <75 and ≥75 years. In SPARTAN, 149 (12%), 741 (61%) and 317 (26%) patients were <65, 65–79 and ≥80 years, and 625 (52%) and 582 (48%) were <75 and ≥75 years. TITAN and SPARTAN patients had broadly similar baseline characteristics across age groups, except that older SPARTAN patients had higher rates of Eastern Cooperative Oncology Group performance status (ECOG PS) of 1, higher median PSA levels and longer median duration of prior ADT for localised disease than younger patients (Tables S1, S2).

### PSA declines

Irrespective of age, rates of confirmed deep PSA declines to ≤0.2 ng/ml were higher with apalutamide plus ADT than ADT alone in both studies (Table 1). In TITAN patients aged <65, 65–79 and ≥80 years, 60%, 71% and 67% from apalutamide and 29%, 33% and 32% from placebo groups achieved PSA ≤ 0.2 ng/ml during the study. Respective numbers in SPARTAN were 48%, 40% and 26% of apalutamide-treated and no placebo-treated patients. In both TITAN and SPARTAN, patients across all age groups treated with apalutamide plus ADT had substantially lower median PSA nadir and longer median time to achieve it versus ADT alone (Table 1).

**Table 1:** Effect of apalutamide treatment on PSA kinetics by age in TITAN (*N* = 1052) and SPARTAN (*N* = 1207) intent-to-treat populations.

	TITAN (mCSPC)						SPARTAN (nmCRPC)					
	<65 yr (*n* = 331)		65–79 yr (*n* = 628)		≥80 yr (*n* = 93)		<65 yr (*n* = 149)		65–79 yr (*n* = 741)		≥80 yr (*n* = 317)	
	APA (*n* = 149)	PBO (*n* = 182)	APA (*n* = 324)	PBO (*n* = 304)	APA (*n* = 52)	PBO (*n* = 41)	APA (*n* = 106)	PBO (*n* = 43)	APA (*n* = 492)	PBO (*n* = 249)	APA (*n* = 208)	PBO (*n* = 109)
PSA nadir^a^												
Median (range), ng/ml	0.04^b^ (0–133)	0.93^c^ (0–408)	0.02^d^ (0–498)	0.68^e^ (0–1408)	0.04 (0–83.7)	0.73 (0–1180)	0.20 (0–48.9)	7.11 (1.5–67.2)	0.32^f^ (0–32.2)	7.18^g^ (0.3–146)	0.75^h^ (0–150)	9.0^i^ (1.0–292)
Median time (range), mo	5.6 (0.1–40.6)	2.8 (0.7–50.6)	5.6 (0.1–47.9)	4.7 (0.8–50.0)	6.4 (1.0–36.8)	3.7 (0.7–40.4)	8.7 (0.9–29.5)	1.0 (0.9–35.7)	7.4^f^ (0.9–44.2)	1.0^g^ (0.2–24.4)	6.5^h^ (0.7–29.4)	1.0^i^ (0.8–8.3)
Confirmed PSA decline ≥50% from baseline^j^												
* n* (%)	132 (89)	86 (47)	292 (90)	180 (59)	49 (94)	24 (59)	98 (93)	1 (2.3)	451 (92)	5 (2.0)	175 (84)	3 (2.8)
Median time^k^ (range), mo	0.95 (0.9–8.3)	0.97 (0.1–30.7)	0.95 (0.3–11.1)	0.99 (0.1–35.4)	0.95 (0.9–9.2)	1.4 (0.9–23.5)	0.95 (0.7–5.2)	20.3 (20.3–20.3)	0.95 (0.5–10.2)	1.9 (1.0–11.2)	0.95 (0.3–6.5)	1.9 (1.8–4.6)
Confirmed PSA decline to ≤0.2 ng/ml^j^												
* n* (%)	90 (60)	52 (29)	231 (71)	101 (33)	35 (67)	13 (32)	51 (48)	0	197 (40)	0	55 (26)	0
Median time^l^ (range), mo	1.9 (0.9–29.4)	1.0 (0.8–39.6)	1.9 (0.1–33.2)	2.8 (0.7–42.0)	1.9 (0.9–25.8)	4.7 (0.9–33.1)	2.8 (0.9–14.9)	NA	2.8 (0.9–25.8)	NA	2.8 (0.3–22.1)	NA

*APA* apalutamide, *mCSPC* metastatic castration-sensitive prostate cancer, *nmCRPC* non-metastatic castration-resistant prostate cancer, *PBO* placebo, *PSA* prostate-specific antigen, *NA* not available due to lack of response.

^a^Patients with available PSA data are included.

^b^*n* = 148.

^c^*n* = 181.

^d^*n* = 321.

^e^*n* = 303.

^f^*n* = 488.

^g^*n* = 246.

^h^*n* = 205.

^I^*n* = 106.

^j^Confirmed by a subsequent measurement ≥4 weeks later.

^k^Assessed in patients with achieved ≥50% PSA decline from baseline.

^l^Assessed in patients with achieved PSA decline to ≤0.2 ng/ml.

### Efficacy outcomes

**Fig. 1:**
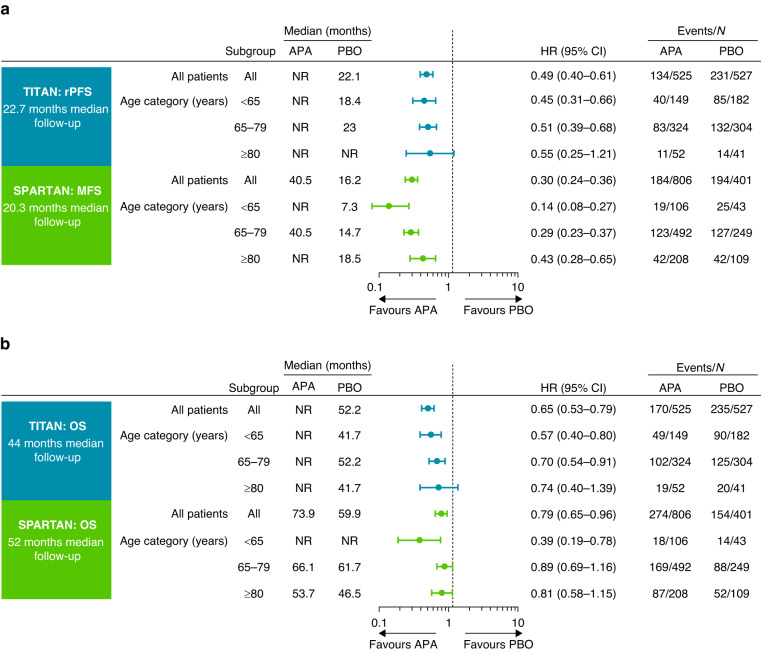
Effect of apalutamide treatment on efficacy endpoints across age groups in TITAN and SPARTAN. **a** Radiographic progression-free survival (TITAN) and metastasis-free survival (SPARTAN). **b** Overall survival. Bars represent 95% CI. APA apalutamide, CI confidence interval, HR hazard ratio, MFS metastasis-free survival, NR not reached, PBO placebo, rPFS radiographic progression-free survival.

In general, patients in all age groups derived benefit with apalutamide across both studies (Fig. 1 and Figs. S2–S4).

In TITAN patients aged <65, 65–79 and ≥80 years, hazard ratios (HRs) (95% confidence interval [CI]) for rPFS were 0.45 (0.31–0.66), 0.51 (0.39–0.68) and 0.55 (0.25–1.21), respectively, favouring apalutamide (Fig. 1a). In respective age groups of SPARTAN patients, HRs (95% CI) for MFS were 0.14 (0.08–0.27), 0.29 (0.23–0.37) and 0.43 (0.28–0.65), also favouring apalutamide. Respective numbers for OS in TITAN were 0.57 (0.4–0.8), 0.70 (0.54–0.91) and 0.74 (0.40–1.39), and those in SPARTAN were 0.39 (0.19–0.78), 0.89 (0.69–1.16) and 0.81 (0.58–1.15) (Fig. 1b); median OS was increased with apalutamide (Fig. S2). PC-specific survival also favoured apalutamide in both studies (Table S3). Results in patients stratified by age <75 versus ≥75 years were similar, consistently favouring apalutamide irrespective of age (Figs. S3, S4). Notably, in the TITAN study, patients aged ≥75 with ECOG PS 1 showed HRs (95% CI) of 0.44 (0.22–0.9) for rPFS and 0.49 (0.26–0.93) for OS. In the SPARTAN study, patients aged ≥75 with ECOG PS 1 demonstrated HRs (95% CI) of 0.42 (0.25–0.71) for MFS and 0.74 (0.49–1.14) for OS. Additionally, in SPARTAN patients aged ≥75 with PSA doubling time (PSADT) ≤ 6 months, HRs (95% CI) for MFS and OS were 0.46 (0.32–0.66) and 0.78 (0.57–1.06), respectively. These findings further support the favourable effects of apalutamide in these patient populations.

### Apalutamide safety profile

In patients aged <65, 65–79 and ≥80 years from both treatment groups, TEAEs occurred in 97%, 97% and 100% in TITAN and in 96%, 96% and 97% in SPARTAN, with similar rates across treatment groups (Table 2). TEAEs leading to discontinuation or death and TEAEs of interest were generally increased with age in both studies regardless of treatment (Tables 2, S4). There was a suggestion of a potential trend towards a greater difference in serious TEAEs and TEAEs leading to discontinuation or death among older adults receiving apalutamide (Table 2).

**Table 2:** Treatment-emergent adverse events in TITAN (*N* = 1051) and SPARTAN (*N* = 1201) safety populations by age^a^.

	TITAN (mCSPC)						SPARTAN (nmCRPC)					
	<65 yr (*n* = 330)		65–79 yr (*n* = 628)		≥80 yr (*n* = 93)		<65 yr (*n* = 149)		65–79 yr (*n* = 739)		≥80 yr (*n* = 313)	
	APA (*n* = 148)	PBO (*n* = 182)	APA (*n* = 324)	PBO (*n* = 304)	APA (*n* = 52)	PBO (*n* = 41)	APA (*n* = 106)	PBO (*n* = 43)	APA (*n* = 491)	PBO (*n* = 248)	APA (*n* = 206)	PBO (*n* = 107)
Median treatment duration (range), mo	39.8 (1.0−52.7)	17.1 (0.5−34.8)	39.8 (0.0−55.7)	21.4 (0.1−37.0)	23.6 (2.0−48.2)	18.9 (0.3−33.1)	46.2 (0.5−69.8)	7.4 (1.4−31.9)	33.4 (0.3−74.5)	11.8 (0.3−37.2)	20.6 (0.1−73.3)	12.0 (0.1−37.1)
Patients with ≥1 TEAE,^b,c^ *n* (%)	142 (96)	177 (97)	316 (98)	292 (96)	52 (100)	41 (100)	104 (98)	39 (91)	475 (97)	231 (93)	202 (98)	103 (96)
Grade 3–4 TEAEs, *n* (%)	70 (47)	82 (45)	155 (48)	114 (38)	34 (65)	24 (59)	54 (51)	14 (33)	266 (54)	90 (36)	129 (63)	41 (38)
Patients with serious TEAEs,^b^ *n* (%)	33 (22)	41 (23)	95 (29)	63 (21)	25 (48)	11 (27)	33 (31)	9 (21)	170 (35)	56 (23)	87 (42)	34 (32)
TEAEs leading to treatment discontinuation, *n* (%)	7 (4.7)	6 (3.3)	42 (13)	18 (5.9)	13 (25)	6 (15)	5 (4.7)	2 (4.7)	61 (12)	14 (5.6)	54 (26)	13 (12)
TEAEs leading to death, *n* (%)	2 (1.4)	2 (1.1)	16 (4.9)	14 (4.6)	2 (3.8)	1 (2.4)	0	0	9 (1.8)	1 (0.4)	15 (7.3)	1 (0.9)
≥1 TEAE of interest, *n* (%)	47 (32)	26 (14)	149 (46)	64 (21)	26 (50)	9 (22)	57 (54)	7 (16)	243 (50)	52 (21)	117 (57)	28 (26)
Skin rash	31 (21)	18 (9.9)	103 (32)	28 (9.2)	19 (37)	3 (7.3)	25 (24)	4 (9.3)	125 (26)	14 (5.6)	62 (30)	7 (6.5)
Fall	7 (4.7)	9 (4.9)	33 (10)	25 (8.2)	9 (17)	3 (7.3)	21 (20)	1 (2.3)	100 (20)	20 (8.1)	56 (27)	17 (16)
Fracture	9 (6.1)	7 (3.8)	38 (12)	19 (6.3)	7 (14)	0	18 (17)	2 (4.7)	90 (18)	16 (6.5)	37 (18)	12 (11)
Ischaemic heart disease	5 (3.4)	0	22 (6.8)	10 (3.3)	4 (7.7)	1 (2.4)	9 (8.5)	1 (2.3)	22 (4.5)	8 (3.2)	13 (6.3)	2 (1.9)
Ischaemic cerebrovascular disorders^d^	1 (0.7)	1 (0.5)	10 (3.1)	5 (1.6)	2 (3.8)	2 (4.9)	NA	NA	NA	NA	NA	NA
Seizure	3 (2.0)	2 (1.1)	0	0	0	0	0	0	2 (0.4)	0	3 (1.5)	0
Total exposure, patient-yr	386	254	863	481	110	58.7	351	37.8	1336	282	431	126
Exposure-adjusted rate, events/100 patient-yr												
Patients with ≥1 TEAE	329	558	393	504	562	504	29.6	103	35.5	81.8	46.9	81.9
Patients with serious TEAEs	11.9	27.5	21.3	18.7	39.9	32.4	9.4	23.8	12.7	19.8	20.2	27.0
TEAEs leading to discontinuation	1.3	2.0	3.9	1.5	10.0	8.5	1.4	5.3	4.1	4.6	10.9	9.5
TEAEs of interest	28.5	20.1	43.3	24.8	58.0	23.9	35.9	39.7	47.7	27.3	75.2	42.1
Skin rash	19.2	10.6	26.4	8.1	27.2	8.5	12.0	26.5	20.4	7.1	25.1	7.2
Fall	2.6	5.5	4.6	7.7	14.5	6.8	7.7	2.6	10.9	7.8	23.0	15.9
Fracture	3.1	2.8	7.0	5.4	10.0	0	9.1	7.9	9.4	6.7	13.7	11.9
Ischaemic heart disease	2.6	0	3.6	2.5	4.5	1.7	3.7	2.6	2.3	3.2	4.2	4.8
Ischaemic cerebrovascular disorders^d^	0.3	0.4	1.7	1.0	1.8	6.8	1.1	0	1.1	0.4	4.9	2.4
Seizure	0.8	0.8	0	0	0	0	0	0	0.1	0	0.7	0

*APA* apalutamide, *mCSPC* metastatic castration-sensitive prostate cancer, *NA* not available, *nmCRPC* non-metastatic castration-resistant prostate cancer, *PBO* placebo, *TEAE* treatment-emergent adverse event.

TEAE rates per 100 patient-years of exposure in each treatment group were calculated as: 100 × distinct events of each preferred term/total patient-year of exposure (total d of exposure/365.25).

^a^One patient in TITAN and six in SPARTAN did not receive study medication.

^b^Patients were counted only once in each category, even if they experienced multiple events in that category.

^c^Excluding grade 5 events.

^d^Ischaemic cerebrovascular disorders in SPARTAN were collected outside of TEAEs of interest.

TITAN and SPARTAN patients from all three age groups were treated for longer with apalutamide than with placebo (Table 2). Exposure-adjusted rates of TEAEs increased with age in both studies regardless of treatment (Table 2); however, TEAEs of interest, including skin rash, falls and fractures, were generally higher in apalutamide- than placebo-treated patients. The use of concomitant bone-sparing medications was similar regardless of treatment, and was observed in 33%, 29% and 41.9% of TITAN and in 32.9%, 31.4% and 39.3% of SPARTAN patients aged <65, 65–79 and ≥80 years, respectively (Table S5). Among patients who had fractures, 10/16, 27/57 and 3/7 TITAN and 9/20, 49/106 and 26/49 SPARTAN patients aged <65, 65–79 and ≥80 years received concomitant bone-sparing medications (Table S5).

### Health-related quality of life

**Fig. 2:**
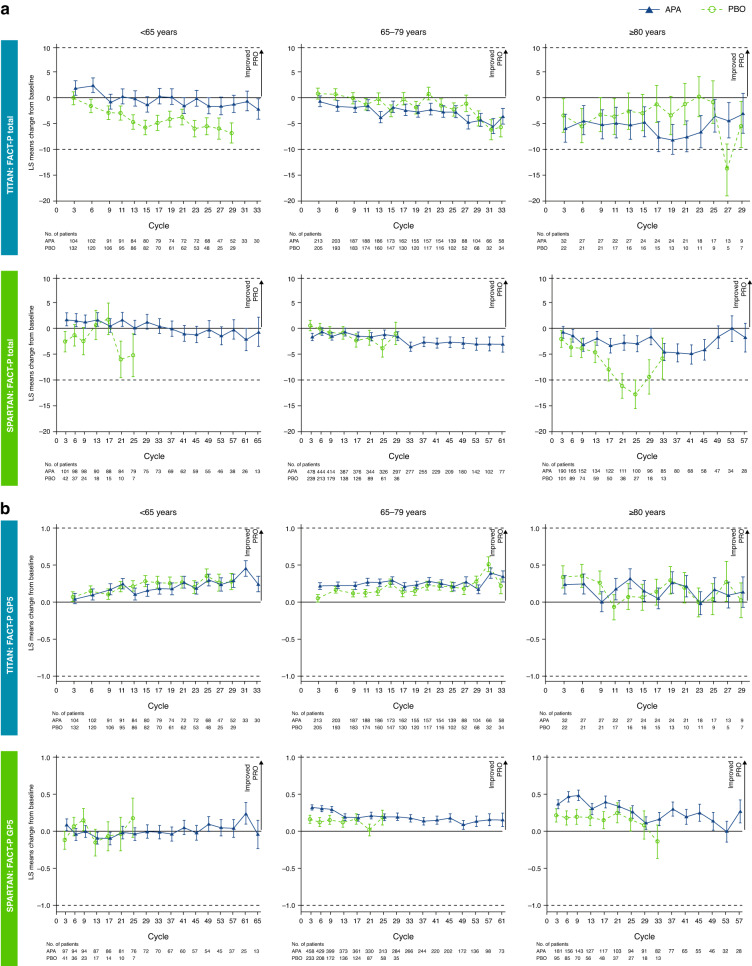
Changes from baseline in patient-reported health-related quality of life in TITAN and SPARTAN, stratified by age. **a** FACT-P total score. **b** FACT-P Physical Wellbeing question GP5: “I am bothered by side effects of treatment”. Bars show standard error, dotted horizontal lines show clinically meaningful change from baseline at visits that had >10% of patients completing FACT-P. APA apalutamide, FACT-P Functional Assessment of Cancer Therapy-Prostate, LS least squares, PBO placebo, PRO patient-reported outcome.

HRQoL in TITAN and SPARTAN was similar across treatment and age groups (Fig. 2).

Apalutamide- and placebo-treated patients across age groups maintained FACT-P total score at similar levels over the course of the study for up to 5.4 and 3 years, respectively, except the oldest SPARTAN placebo-treated patients, who reported lower FACT-P than patients receiving apalutamide (Fig. 2a). Regardless of age, apalutamide-treated patients reported not being bothered by treatment side effects over time or not any more than placebo-treated patients (Fig. 2b). The overall physical wellbeing and energy levels remained consistent over time in all age groups of apalutamide-treated patients from both studies and were comparable with those in placebo-treated patients (Fig. S5). Similar results for FACT-P total score were observed in patients aged <75 or ≥75 years (Fig. S6). TITAN and SPARTAN patients aged ≥75 years with ECOG PS of 1 at baseline or SPARTAN patients with PSADT ≤ 6 months have maintained general HRQoL per FACT-P total score (data not shown).

## Discussion

In this post hoc analysis of TITAN and SPARTAN, efficacy generally favoured apalutamide plus ADT versus ADT alone across all age groups. The safety profile of apalutamide was consistent with previous reports [2, 7, 18, 19], and patients from all age groups tolerated apalutamide treatment well. There was a suggestion of a potential trend towards a greater difference in serious TEAEs and TEAEs leading to discontinuation or death among older adults receiving apalutamide.

Apalutamide showed greater disease control per PSA kinetics, regardless of age, than placebo. Thus, TITAN patients across all age groups achieved lower median PSA nadir with apalutamide plus ADT versus ADT alone, and the majority achieved deep PSA decline to ≤0.2 ng/ml, consistent with the overall population [25]. SPARTAN patients also achieved lower PSA nadir with apalutamide plus ADT versus ADT alone regardless of age. A large proportion of apalutamide-treated and no placebo-treated SPARTAN patients achieved deep PSA decline to ≤0.2 ng/ml across all age groups, consistent with the overall population [26]. In both studies, time to achieve PSA nadir with apalutamide plus ADT was longer than that with ADT alone, consistent with lower PSA nadir and the overall populations [25, 26]. Notably, PSA nadir values and rate of PSA ≤ 0.2 ng/ml decreased with age in SPARTAN. Approximately 50% of younger (<65 years) and a quarter of octogenarian (≥80 years) apalutamide-treated patients achieved PSA ≤ 0.2 ng/ml at any time during the study, whereas in the overall population this rate was 38% [26]. Diminishing PSA control may be associated with more aggressive disease in SPARTAN patients aged 65–79 and ≥80 years who had longer time on prior ADT and higher PSA levels at baseline than younger patients.

Regardless of patient age, apalutamide also showed a clinical benefit consistent with previous findings [2, 7, 18, 19]. Apalutamide treatment effect on rPFS and OS in TITAN was seen across all age groups but was most pronounced in patients aged <65 and 65–79 years. The ≥80 year subgroup had seemingly less pronounced benefits but had a relatively small number of patients. With the dichotomous cutoff of 75 years, the subgroup of TITAN patients aged ≥75 years was larger, and rPFS and OS benefits from apalutamide were reassuringly consistent with those in the younger subgroup. In SPARTAN patients, treatment effect of apalutamide for MFS was observed regardless of age. The OS benefit was most pronounced in the youngest patients (<65 years), likely owing to less aggressive disease or decreased frailty as reflected by low PSA levels and low ECOG PS score at baseline, and also reflecting longer apalutamide exposure. Nevertheless, OS favoured apalutamide in older SPARTAN patients.

The overall safety profile of apalutamide in TITAN and SPARTAN was consistent with previous reports [18, 19]. Rash, known to be more common in apalutamide-treated than placebo-treated patients [18, 19], increased with age, consistent with the age-related susceptibility to toxic complications [27]. Rates of falls and fractures also increased with age in both treatment groups of TITAN and SPARTAN, consistent with age- and ADT-related falls and osteoporosis reported previously [11, 12, 27]. Falls and fractures were more frequent with apalutamide than with ADT, supporting our previous finding that older age was one of the independent predictors of falls in apalutamide-treated SPARTAN patients [28]. Falls and fractures were also shown to be associated with enzalutamide and age in nmCRPC [13]. A prevention programme should be in place to address falls and fractures in patients with advanced PC during oncologic treatments. Interestingly, the use of bone-sparing medications in TITAN and SPARTAN was <40% and did not consistently increase with age. Only ≈40–50% of TITAN and SPARTAN patients aged >65 years who had fractures received bone-sparing medications, suggesting inadequate addressing of bone-related syndromes in older patients with advanced disease. It should be recognised that older adults with cancer are at higher risk for falls than the general geriatric population [29, 30]. Current guidelines recommend denosumab or zoledronic acid mainly in mCRPC [30]; however, they should also be considered in patients with primary or developing osteopenia or osteoporosis regardless of prostate disease state.

Despite an increase in several types of AEs with addition of apalutamide to ADT, patients of all ages reported minimal side effect bother and no increase in fatigue. In all age groups, there were no clinically meaningful differences in overall HRQoL between apalutamide- and placebo-treated patients. Among patients aged >80 years with nmCRPC, there was a notable trend toward longer maintenance of HRQoL with apalutamide plus ADT versus ADT alone.

Our analysis supports early treatment of patients with advanced disease with apalutamide, regardless of age. PSA decline and long-term survival in TITAN and SPARTAN favoured apalutamide across all age groups but became less pronounced with older age in SPARTAN, likely due to higher median age in SPARTAN (74 years [7]) than in TITAN (69 years [2]). Age-related toxicity and syndromes known in older patients [27] may explain the increased susceptibility of older TITAN and SPARTAN patients to rash and predispose them to falls and fractures, also emphasising the need to address these concurrently.

Limitations of this analysis include its post hoc nature and small size of some subgroups. Furthermore, there is variability in group sizes and potential imbalances in co-morbidities, which were not accounted for in this analysis. Prospective studies with larger populations of older patients are needed. Despite the limitations, our analysis supports apalutamide benefit for older patients with advanced PC.

## Conclusions

This post hoc analysis of TITAN and SPARTAN patients with mCSPC and nmCRPC demonstrates a benefit with apalutamide regardless of patient age. The tolerability of apalutamide varied across age groups, with more notable differences in older adults. Known AEs associated with apalutamide should be considered in the context of age and addressed accordingly with individualised treatment based on disease stage, physiological status and patient preference.

### Supplementary information

Shen TITAN/SPARTAN Older Pts_Supplement

## Data Availability

The data sharing policy of Janssen Pharmaceutical Companies of Johnson & Johnson is available at https://www.janssen.com/clinical-trials/transparency. As noted on this site, requests for access to the study data can be submitted through Yale Open Data Access (YODA) Project site at http://yoda.yale.edu.
