# Supplementary material for: Apalutamide efficacy, safety and wellbeing in older patients with advanced prostate cancer from Phase 3 randomised clinical studies TITAN and SPARTAN
Source: Br J Cancer. 2023 Nov 11;130(1):73–81. doi: 10.1038/s41416-023-02492-8 (PMC10781967; doi:10.1038/s41416-023-02492-8)

SUPPLEMENTARY MATERIAL

Apalutamide efficacy, safety and wellbeing in older patients with advanced prostate cancer from Phase 3 randomised clinical studies TITAN and SPARTAN

John Shen, Simon Chowdhury, Neeraj Agarwal, Lawrence I. Karsh, Stéphane Oudard, Benjamin A. Gartrell, Susan Feyerabend, Fred Saad, Christopher M. Pieczonka, Kim N. Chi, Sabine D. Brookman-May, Brendan Rooney, Amitabha Bhaumik, Sharon A. McCarthy, Katherine B. Bevans, Suneel D. Mundle, Eric J. Small, Matthew R. Smith, Julie N. Graff

SUPPLEMENTARY METHODS

TITAN and SPARTAN study designs

TITAN enrolled 1052 patients diagnosed with metastatic castration-sensitive prostate cancer (mCSPC) with at least one bone metastasis. Patients were recruited at primary, secondary and tertiary health care and community centres, and were stratified according to Gleason score at diagnosis (≤7 vs >7, on a scale of 2–10, with higher scores indicating higher-grade cancer that may be more aggressive), geographic region (North America and European Union vs all other countries) and previous treatment with docetaxel (yes vs no), and were then randomised 1:1 to receive oral apalutamide 240 mg/day (n = 525) or placebo (n = 527) added to concurrent androgen-deprivation therapy (ADT). The interactive web response system (IWRS) randomly balanced stratification criteria and generated a unique treatment code that dictated patients' treatment, using randomly permuted blocks. The patients, staff and sponsor representatives were unaware of treatment allocations. IWRS maintained generated patient codes.

SPARTAN enrolled 1207 patients with non-metastatic castration-resistant prostate cancer (nmCRPC) who had high risk of distant metastasis, defined by prostate-specific antigen (PSA) doubling time ≤10 months despite continuous ADT. Patients were recruited at primary, secondary and tertiary health care and community centres, and were stratified according to PSA doubling time (>6 months vs ≤6 months), use of bone-sparing agents (yes vs no) and classification of local or regional nodal disease (N0 vs N1) at the time of study entry, and then were randomised 2:1 to receive oral apalutamide 240 mg/day (n = 806) or placebo (n = 401) plus ongoing ADT. The interactive voice randomisation system assigned patients to treatment and balance stratification criteria. The patients, staff and sponsor representatives were unaware of the patients' PSA values and group assignments until unblinding occurred. All randomisation codes and data sets identified by a coded number were stored in separate locked file cabinets in a secure area not accessible to individuals related to the protocol.

Outcomes

Dual primary endpoints of TITAN were radiographic progression-free survival (rPFS) and overall survival (OS). rPFS was defined as time from randomisation to first documented progressive disease by conventional imaging or death. The study assessed conventional imaging based on Response Evaluation Criteria In Solid Tumors (RECIST), version 1.1,[1] modified by Prostate Cancer Working Group 2 criteria.[2] OS was defined as the time from randomisation to the date of death from any cause. TITAN secondary endpoints were the time to cytotoxic chemotherapy defined as the time from randomisation to initiation of cytotoxic chemotherapy, time to pain progression as assessed by average increase in 2 points from baseline in Brief Pain Inventory–Short Form, time to chronic opioid use defined as the time from randomisation to chronic opioid use, and time to skeletal-related event defined as the time from randomisation to first observation of a skeletal-related event (symptomatic pathologic fracture, spinal cord compression, radiation to bone or surgery to bone). Patient-reported outcomes (PROs) for health-related quality of life (HRQoL) were assessed by means of the Functional Assessment of Cancer Therapy-Prostate (FACT-P) questionnaire.

The primary endpoint of SPARTAN was metastasis-free survival (MFS), defined as time from randomisation until the first documented distant metastasis by conventional imaging or death. The study assessed conventional imaging based on RECIST, version 1.1.[1]

SPARTAN secondary endpoints were time to metastasis (defined as the time from randomisation to the first detection of distant metastasis involving the bone or soft tissue on conventional imaging, as assessed by means of blinded independent central review), progression-free survival (defined as the time from randomisation to the first detection of local or distant metastatic disease on conventional imaging, as assessed by means of blinded independent central review, or death from any cause, whichever occurred first), time to symptomatic progression (defined as the time from randomisation to a skeletal-related event, pain progression or worsening of disease-related symptoms leading to the initiation of a new systemic anticancer therapy or the time to the development of

clinically significant symptoms due to local or regional tumour progression leading to surgery or radiation therapy), OS and time to the initiation of cytotoxic chemotherapy. PROs were assessed with the FACT-P questionnaire and the three-level version of the European Quality of Life–5 Dimensions (EQ-5D-3L) questionnaire.

Eligibility criteria

Eligible TITAN patients were required to have documented adenocarcinoma of the prostate and distant metastatic disease per conventional imaging with or without visceral or lymph node involvement. TITAN patients were castration sensitive (i.e., they were not receiving ADT at the time of disease progression).

Eligible SPARTAN patients had no distant metastases per conventional imaging, with N0 disease or N1 disease if malignant pelvic lymph nodes were measured <2 cm in the short axis and were located below the aortic bifurcation. SPARTAN patients were castration resistant and at high risk for the development of metastasis, which was defined as a PSA doubling time of 10 months or less during continuous ADT.

PSA assessment

In TITAN, serum PSA was assessed at screening, on day 1 of each treatment cycle until cycle 13, every other cycle until cycle 25, and every four cycles until the end-of-treatment visit. In SPARTAN, PSA was assessed at screening, on day 1 of every cycle from cycle 1 to 6, every other cycle from cycle 7 to 13, and every four cycles until end of treatment and at final follow-up.

Safety assessment

Treatment-emergent adverse events (TEAEs) were defined as AEs that occurred on or after first dose of the study drug through one cycle, defined as 30 days in TITAN and 28 days in SPARTAN, after the last study treatment. TEAEs were coded using *Medical Dictionary for Regulatory Activities* versions 20.0 (TITAN) and 19.1 (SPARTAN) and graded according to the National Cancer Institute Common Terminology Criteria for Adverse Events version 4.03 (TITAN) and 4.0 (SPARTAN). Concomitant bone-sparing

medications from the start of study treatments until 30 or 28 days after the last dose (for TITAN and SPARTAN, respectively) were analysed as the World Health Organization Anatomical Therapeutic Chemical Level 2 code “Drugs for treatment of bone diseases” and Level 4 codes “Vitamin D and analogues” or “Vitamin A and D in combination”.

Sample size determination

For TITAN dual primary endpoints of rPFS and OS, an overall type I error of 5% was planned with the level of significance of 0.005 allocated for rPFS and of 0.045 for OS. The study was considered a success if at least one of the two primary endpoints was statistically significant. The study was designed to provide ≥85% power to detect 33% reduction in the hazard of radiographic progression or death and ≈80% power to detect a 25% reduction in the hazard of death for patients receiving apalutamide. For the preplanned final updated OS analysis in TITAN, 410 events were required to detect the above reduction at a two-tailed significance level of 0.045.

In SPARTAN, the primary endpoint was designed to provide 90% power to detect 30% reduction in the risk of developing metastases for patients receiving apalutamide with two-tailed significance level of 0.05. The final OS analysis was scheduled to occur after 427 deaths with ≈80% power to detect a 25% reduction in the hazard of death, and two-tailed significance level of 0.05. AEs were collected throughout the studies.

Supplementary reference

1. Eisenhauer EA, Therasse P, Bogaerts J, et al. New response evaluation criteria in solid tumours: revised RECIST guideline (version 1.1). *Eur J Cancer* **45**, 228–247 (2009).

2. Scher HI, Halabi S, Tannock I, et al. Design and end points of clinical trials for patients with progressive prostate cancer and castrate levels of testosterone: recommendations of the Prostate Cancer Clinical Trials Working Group. *J Clin Oncol* **26**, 1148–1159 (2008).

Table S1. Baseline characteristics of TITAN mCSPC ITT population (*N* = 1052) by <65, 65–79 and ≥80 year age groups.

	**<65 yr (*n* = 331)**		**65–79 yr (*n* = 628)**		**≥80 yr (*n* = 93)**	
	**APA (*n* = 149)**	**PBO (*n* = 182)**	**APA (*n* = 324)**	**PBO (*n* = 304)**	**APA (*n* = 52)**	**PBO (*n* = 41)**
ECOG PS score						
0	88 (59)	118 (65)	212 (65)	201 (66)	28 (54)	29 (71)
1	61 (41)	64 (35)	112 (35)	103 (34)	24 (46)	12 (29)[a]
Gleason score at first diagnosis						
≤7	43 (29)	50 (28)	115 (36)	109 (36)	16 (31)	10 (24)
≥8	106 (71)	132 (73)	209 (65)	195 (64)	36 (69)	31 (76)
Metastatic stage at first diagnosis						
M0	16 (11)	16 (8.8)	55 (17)	36 (12)	14 (27)	7 (17)
M1	123 (83)	161 (89)	251 (78)	251 (83)	37 (71)	29 (71)
MX	10 (6.7)	5 (2.7)	18 (5.6)	17 (5.6)	1 (1.9)	5 (12)
Disease volume						
Low	52 (35)	57 (31)	130 (40)	119 (39)	18 (35)	16 (39)
High	97 (65)	125 (69)	194 (60)	185 (61)	34 (65)	25 (61)

Prior docetaxel treatment	25 (17)	32 (18)	31 (9.6)	21 (6.9)	2 (3.8)	2 (4.9)
Prior ADT for localised prostate cancer	5 (3.4)	8 (4.4)	22 (6.8)	13 (4.3)	4 (7.7)	3 (7.3)
Median duration (range), mo	2.8 (0–25.8)	3.0 (0–32.3)	9.2 (0–33.6)	12.0 (0–36.0)	2.1 (0.5–12.0)	5.2 (3.9–20.8)
Median PSA level (range), ng/ml	5.8 (0–2256)	4.5 (0–2229)	6.9 (0–2682)	3.7 (0–1408)	4.6 (0.1–1111)	6.3 (0–1180)
Number of bone lesions						
≤10	87 (58)	106 (58)	196 (61)	198 (65)	35 (67)	27 (66)
>10	62 (42)	76 (42)	128 (40)	106 (35)	17 (33)	14 (34)
Extent of disease						
Bone only	80 (54)	91 (50)	183 (57)	155 (51)	26 (50)	23 (56)
Bone + lymph node	58 (39)	83 (46)	122 (38)	121 (40)	19 (37)	15 (37)
Bone + lung	13 (8.7)	18 (9.9)	24 (7.4)	42 (14)	10 (19)	4 (9.8)
Bone + liver	7 (4.7)	6 (3.3)	5 (1.5)	5 (1.6)	0	2 (4.9)
Bone + soft tissue	6 (4.0)	5 (2.7)	12 (3.7)	22 (7.2)	4 (7.7)	0

ADT androgen-deprivation therapy, *APA* apalutamide, *ECOG PS* Eastern Cooperative Oncology Group performance status, *ITT* intent to treat, *mCSPC* metastatic castration-sensitive prostate cancer, *mHSPC* metastatic hormone-sensitive prostate cancer, *PBO* placebo, *PSA* prostate-specific antigen.

Values are *n* (%) unless noted.

[a]One patient had an ECOG PS score of 2.

Table S2. Baseline characteristics of SPARTAN nmCRPC ITT population (*N* = 1207) by <65, 65–79 and ≥80 year age groups.

	**<65 yr (*n* = 149)**		**65–79 yr (*n* = 741)**		**≥80 yr (*n* = 317)**	
	**APA (*n* = 106)**	**PBO (*n* = 43)**	**APA (*n* = 492)**	**PBO (*n* = 249)**	**APA (*n* = 208)**	**PBO (*n* = 109)**
ECOG PS score						(*n* =108)
0	96 (91)	39 (91)	397 (81)	202 (81)	130 (63)	70 (65)
1	10 (9.4)	4 (9.3)	95 (19)	47 (19)	78 (38)	38 (35)
Gleason score at first diagnosis			(*n* = 483)	(n = 243)	(*n* = 195)	(*n* = 101)
≤7	57 (54)	21 (49)	266 (55)	129 (53)	120 (62)	68 (67)
≥8	49 (46)	22 (51)	217 (45)	114 (47)	75 (39)	33 (33)
Tumour stage at first diagnosis		(n = 42)	(*n* = 486)	(*n* = 246)	(*n* = 202)	(*n* = 106)
T1	16 (15)	1 (2.4)	75 (15)	38 (15)	50 (25)	24 (23)
T2	31 (29)	11 (26)	174 (36)	72 (29)	60 (30)	40 (38)
T3	50 (47)	25 (60)	186 (38)	110 (45)	60 (30)	28 (26)
T4	3 (2.8)	3 (7.1)	20 (4.1)	11 (4.5)	9 (4.5)	2 (1.9)
TX	6 (5.7)	2 (4.8)	31 (6.4)	15 (6.1)	23 (11)	12 (11)

Lymph node stage at first diagnosis			(*n* = 488)	(*n* = 246)	(*n* = 205)	(*n* = 106)
N0	66 (62)	21 (49)	336 (69)	178 (72)	148 (72)	74 (70)
N1	33 (31)	17 (40)	65 (13)	40 (16)	20 (9.8)	4 (3.8)
NX	7 (6.6)	5 (12)	87 (18)	28 (11)	37 (18)	28 (26)
Median duration of ADT (range), mo	(*n* = 87)	(*n* = 35)	(*n* = 401)	(*n* = 195)	(*n* = 172)	(*n* = 91)
	27.0 (0–142)	20.5 (0.2–99)	32.9 (0–213)	28.8 (0–186)	41.3 (1.7–184)	36.6 (0.1–196)
Median PSA level (range), ng/ml	6.3 (0.4–167)	7.7 (1.5–67.2)	7.8 (0.1–295)	7.5 (1.1–146)	8.6 (0.5–210)	10.3 (1.9–292)
Median PSA doubling time, mo (range)	3.6 (0.8–9.7)	2.9 (0.7–9.7)	4.2 (0.8–10.0)	4.5 (0.7–10.0)	5.1 (1.2–10.0)	4.7 (1.3–9.7)

ADT androgen-deprivation therapy, *APA* apalutamide, *ECOG PS* Eastern Cooperative Oncology Group performance status, *ITT* intent to treat, *nmCRPC* non-metastatic castration-sensitive prostate cancer, *PBO* placebo, *PSA* prostate-specific antigen.

Values are *n* (%) unless noted.

Table S3. Prostate cancer-specific survival across age groups.

	TITAN (mCSPC)					SPARTAN (nmCRPC)				
**Outcome**	**Apalutamide + ADT**		**Placebo + ADT**		**HR (95% CI)**	**Apalutamide + ADT**		**Placebo + ADT**		**HR (95% CI)**
	**Events, *n***	**Patients at risk, *n***	**Events, *n***	**Patients at risk, *n***		**Events, *n***	**Patients at risk, *n***	**Events, *n***	**Patients at risk, *n***	
Overall population	115	525	166	527	0.62 (0.49–0.79)	158	806	94	401	0.75 (0.58–0.96)
<65 yr	73	182	40	149	0.57 (0.38–0.83)	14	106	10	43	0.41 (0.18–0.91)
65–79 yr	82	304	62	324	0.65 (0.47–0.90)	111	492	53	249	0.98 (0.7–1.35)
≥80 yr	11	41	13	52	0.93 (0.42–2.07)	33	208	31	109	0.52 (0.32–0.85)

ADT androgen-deprivation therapy, *CI* confidence interval, *HR* hazard ratio, *mCSPC* metastatic castration-sensitive prostate cancer, *nmCRPC* non-metastatic castration-resistant prostate cancer.

Table S4. Treatment-emergent adverse events in TITAN (*N* = 1051) and SPARTAN (*N* = 1201) safety populations by <75 years versus ≥75 years.

	**TITAN (mCSPC)**				**SPARTAN (nmCRPC)**			
	**<75 yr**		**≥75 yr**		**<75 yr**		**≥75 yr**	
	**APA (*n* = 391)**	**PBO (*n* = 414)**	**APA (*n* = 133)**	**PBO (*n* = 113)**	**APA (*n* = 412)**	**PBO (*n* = 211)**	**APA (*n* = 391)**	**PBO (*n* = 187)**
Median treatment duration (range), mo	39.8 (0.2–55.7)	19.9 (0.1–37.0)	33.7 (0–54.5)	20.9 (0.3–35.3)	37.9 (0.3–74.5)	11.1 (0.4–36.8)	25.8 (0.1–73.3)	14.7 (0.1–37.2)
Patients with ≥1 TEAE[a,b]	377 (96)	398 (96)	133 (100)	112 (99)	398 (97)	198 (94)	383 (98)	175 (94)
Grade 3–4 TEAEs	188 (48)	163 (39)	71 (53)	57 (50)	213 (52)	72 (34)	236 (60)	73 (39)
Patients with SAEs[a]	101 (26)	86 (21)	52 (39)	29 (26)	134 (33)	41 (19)	156 (40)	58 (31)
Any TEAE leading to treatment discontinuation	37 (9.5)	17 (4.1)	25 (19)	13 (12)	38 (9.2)	10 (4.7)	82 (21)	19 (10)
TEAEs leading to death	16 (4.1)	10 (2.4)	4 (3.0)	7 (6.2)	4 (1.0)	0	20 (5.1)	2 (1.1)
≥1 TEAE of interest	153 (39)	71 (17)	69 (52)	28 (25)	195 (47)	35 (17)	222 (57)	52 (28)
Skin rash	103 (26)	36 (8.7)	50 (38)	13 (12)	94 (23)	12 (5.7)	118 (30)	13 (7.0)
Fracture	34 (8.7)	22 (5.3)	20 (15)	4 (3.5)	67 (16)	9 (4.3)	78 (20)	21 (11)
Fall	30 (7.7)	26 (6.3)	19 (14)	11 (9.7)	75 (18)	11 (5.2)	102 (26)	27 (14)
Ischaemic heart disease	18 (4.6)	7 (1.7)	13 (9.8)	4 (3.5)	18 (4.4)	6 (2.8)	26 (6.6)	5 (2.7)

Ischaemic cerebrovascular disorders[c]	8 (2.0)	4 (1.0)	5 (3.8)	4 (3.5)	NA	NA	NA	NA
Seizure	3 (0.8)	2 (0.5)	0	0	0	0	5 (1.3)	0

APA apalutamide, *mCSPC* metastatic castration-sensitive prostate cancer, *NA* not available, *nmCRPC* non-metastatic castration-resistant prostate cancer, *PBO* placebo, *SAE* serious adverse event, *TEAE* treatment-emergent adverse event.

Values are n (%) unless noted.

[a]Patients were counted only once in each category, even if they experienced multiple events in that category.

[b]Excluding grade 5 events.

[c]Ischaemic cerebrovascular disorders in SPARTAN were collected outside of TEAEs of interest.

Table S5. Concomitant bone-sparing agents in TITAN (*N* = 1051) and SPARTAN (*N* = 1201) safety populations <65, 65–79 and ≥80 year age groups.

Data values show number (%)	TITAN (mCSPC)						SPARTAN (nmCRPC)					
	<65 yr		65–79 yr		≥80 yr		<65 yr		65–79 yr		≥80 yr	
	APA (*n* = 148)	PBO (*n* = 182)	APA (*n* = 324)	PBO (*n* = 304)	APA (*n* = 52)	PBO (*n* = 41)	APA (*n* = 106)	PBO (*n* = 43)	APA (*n* = 491)	PBO (n = 248)	APA (*n* = 206)	PBO (*n* = 107)
≥1 concomitant bone-sparing medication	45 (30)	64 (35)	89 (28)	93 (31)	23 (44)	16 (39)	39 (37)	10 (23)	159 (32)	73 (29)	83 (40)	40 (37)
Drugs to treat bone diseases[a,b]	40 (27)	50 (28)	55 (17)	69 (23)	9 (17)	9 (22)	11 (10)	5 (12)	64 (13)	29 (12)	39 (19)	14 (13)
Vitamins[a]												
Vitamin D and analogues[c,d]	10 (6.8)	27 (15)	42 (13)	36 (12)	19 (37)	12 (29)	30 (28)	7 (16)	121 (25)	53 (21)	57 (28)	34 (32)
Vitamin A and D combination[c,e]	0	0	0	3 (1.0)	0	0	2 (1.9)	1 (2.3)	5 (1.0)	0	2 (1.0)	2 (1.9)
≥1 concomitant bone-sparing medication and treatment-emergent fractures	5 (3.4)	5 (2.7)	19 (5.9)	8 (2.6)	3 (5.8)	0	8 (7.5)	1 (2.3)	43 (8.8)	6 (2.4)	19 (9.2)	7 (6.6)
Drugs to treat bone diseases[a]	4 (2.7)	4 (2.2)	11 (3.4)	4 (1.3)	2 (3.8)	0	2 (1.9)	0	25 (5.1)	2 (0.8)	11 (5.3)	2 (1.9)

Vitamins[a]	2 (1.4)	3 (1.6)	10 (3.1)	4 (1.3)	3 (5.8)	0	7 (6.6)	1 (2.3)	32 (6.5)	5 (2.0)	13 (6.3)	6 (5.7)
Vitamin D and analogues[c]	2 (1.4)	3 (1.6)	10 (3.1)	4 (1.3)	3 (5.8)	0	7 (6.6)	1 (2.3)	31 (6.3)	5 (2.0)	13 (6.3)	6 (5.7)
Vitamin A and D combination[c]	0	0	0	0	0	0	0	0	1 (0.2)	0	0	0

APA apalutamide, *mCSPC* metastatic castration-sensitive prostate cancer, *nmCRPC* non-metastatic castration-resistant prostate cancer, *PBO* placebo.

Recurrent medications are counted only once per patient.

[a]World Health Organization Anatomical Therapeutic Code Level 2.

[b]Includes bisphosphonates (alendronic acid, clodronic acid, disodium clodronate, disodium pamidronate, ibandronic acid, minodronic acid, risedronic acid, sodium alendronate, sodium ibandronate, sodium risedronate, and zoledronic acid), other drugs affecting bone structure and mineralisation (denosumab, collagen hydroxyapatite complex and strontium ranelate) and bisphosphonate combinations (Fosavance) in SPARTAN. Includes bisphosphonates (alendronic acid, clodronate, pamidronate, ibandronic acid, risedronic acid, sodium alendronate, sodium ibandronate, sodium risedronate and zoledronic acid), other drugs affecting bone structure and mineralisation (denosumab and collagen-hydroxyapatite complex), bisphosphonate combinations (Fosavance) and uncoded in TITAN.

[c]World Health Organization Anatomical Therapeutic Code Level 4.

[d]Includes alfacalcidol, calcifediol, calcitriol, colecalciferol, ergocalciferol, eldecalcitol and vitamin D NOS in SPARTAN, and alfacalcidol, calcifediol, calcitriol, ergocalciferol, eldecalcitol and vitamin D NOS in TITAN.

[e]Includes cod liver oil, retinol with vitamin D NOS and Vitamidyne A and D or 00343801 in SPARTAN, and cod liver oil in TITAN.

Fig. S1 TITAN and SPARTAN patient flows. (a) TITAN. **(b)** SPARTAN. [a]One patient from each group was lost to follow-up. ADT androgen-deprivation therapy, ITT intent-to-treat.

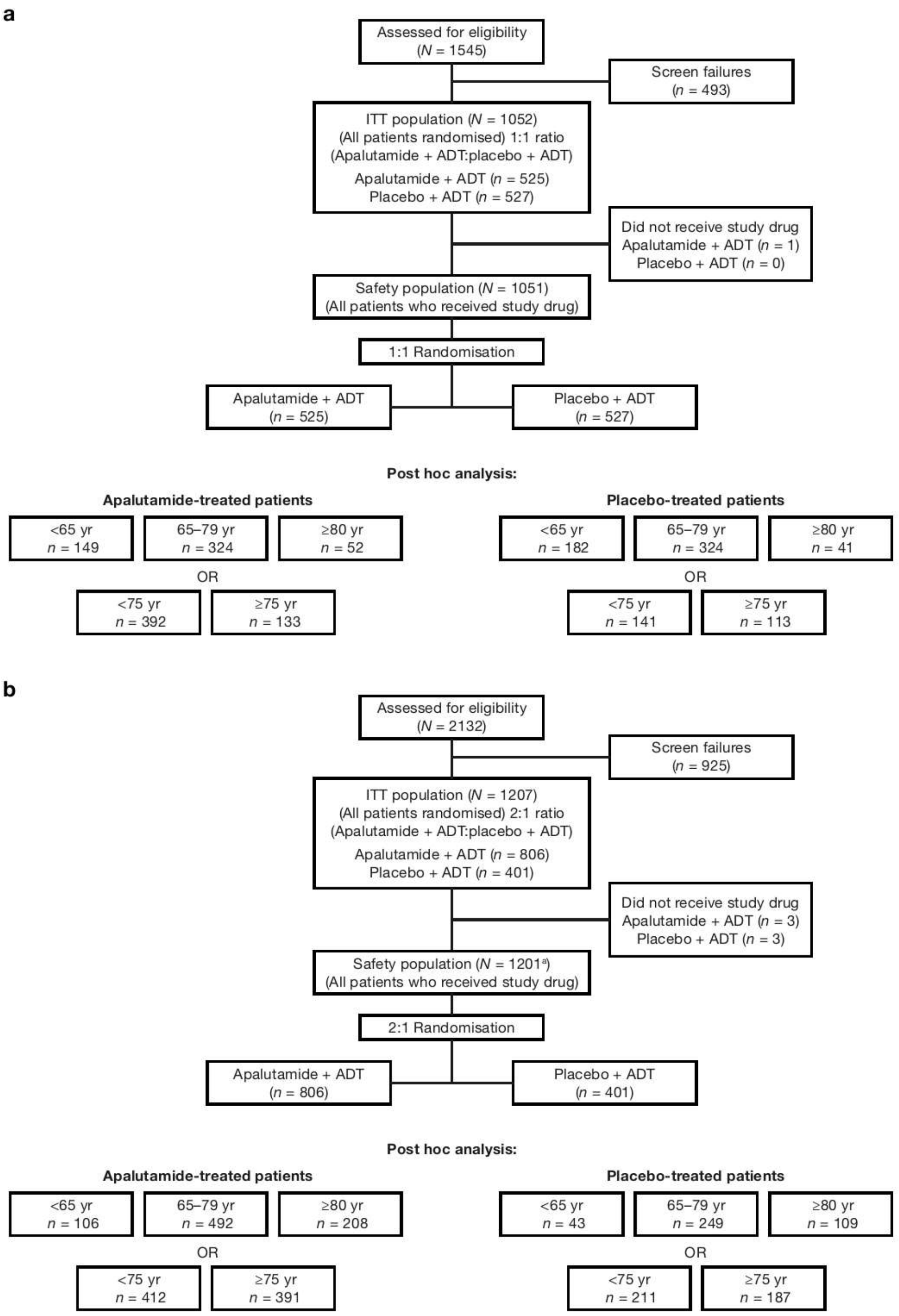

Fig. S2 Kaplan–Meier estimates of OS in TITAN and SPARTAN patients by <65, 65–79 and ≥80 year age groups. The curves are truncated for instances in which the number at risk in a group was <5. APA apalutamide, OS overall survival, PBO placebo, rPFS radiographic progression-free survival.

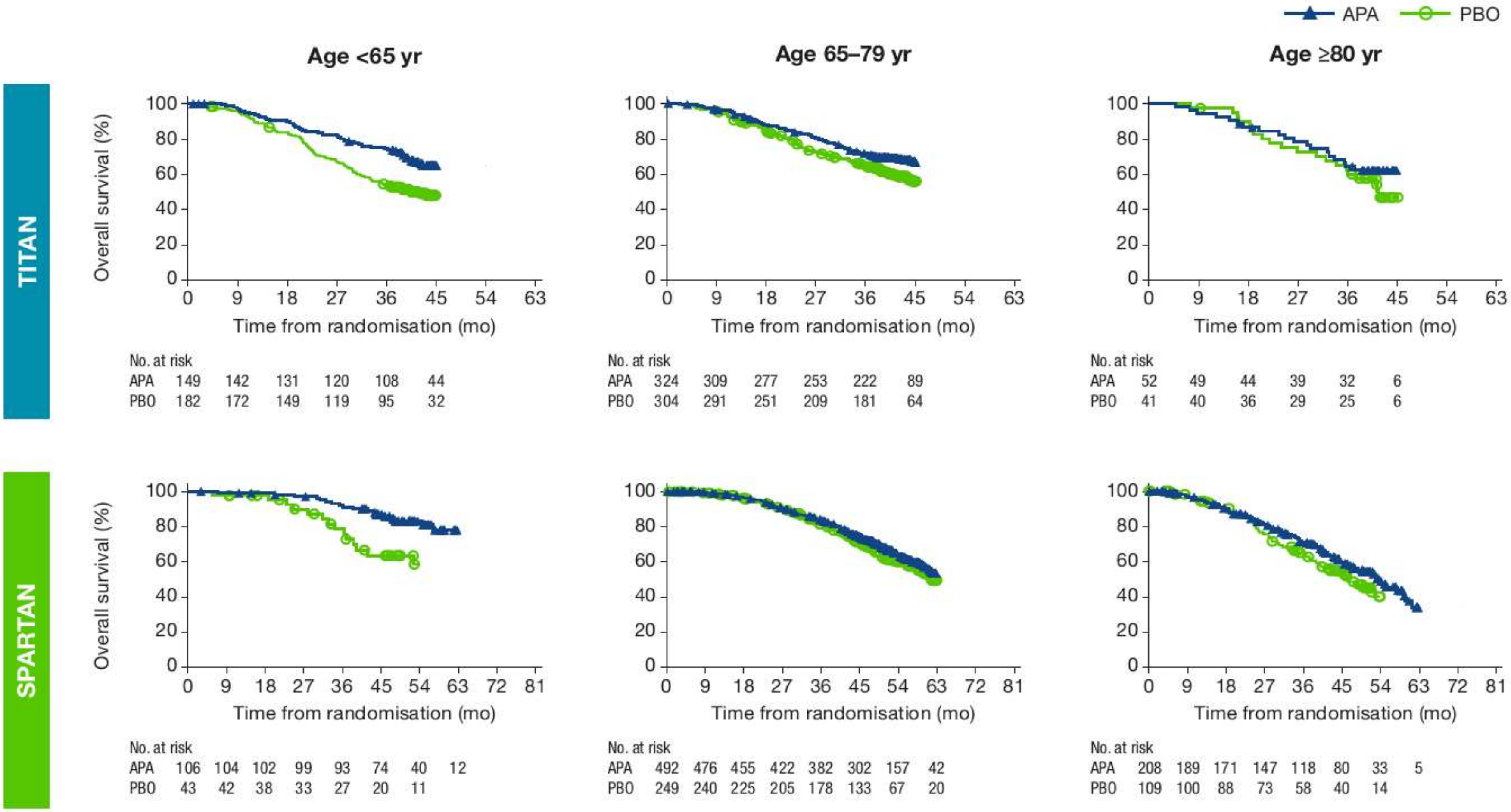

Fig. S3 TITAN efficacy endpoints by age <75 years versus ≥75 years. (a) Dual primary endpoint of rPFS; median follow-up 22.7 months. **(b)** Dual primary endpoint of OS; median follow-up of 44.0 months. Bars represent 95% CI. APA apalutamide, CI confidence interval, HR hazard ratio, NR not reached, OS overall survival, PBO placebo, rPFS radiographic progression-free survival.

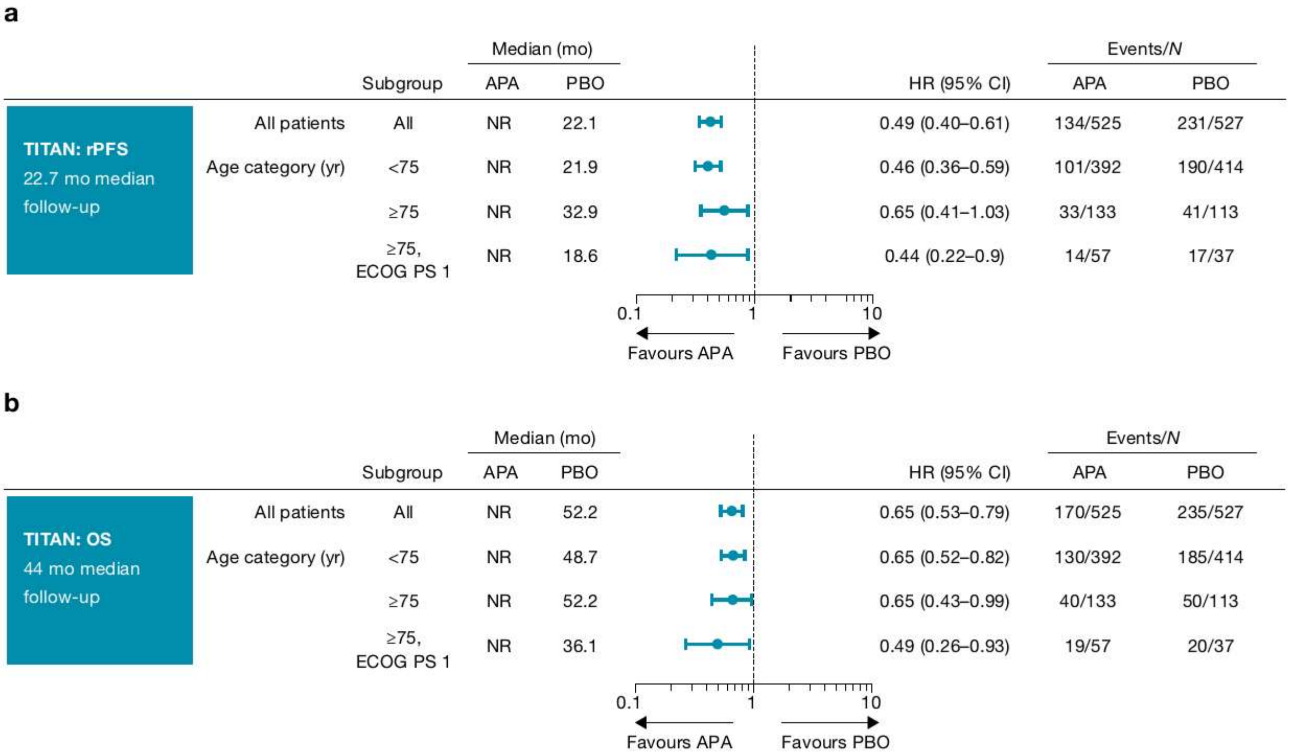

Fig. S4 SPARTAN efficacy endpoints stratified by age <75 years versus ≥75 years. (a) Primary endpoint of MFS; median follow-up of 22.3 months. **(b)** OS; median follow-up 52 months. Bars represent 95% CI. APA apalutamide, CI confidence interval, HR hazard ratio, MFS metastasis-free survival, NR not reached, OS overall survival, PBO placebo.

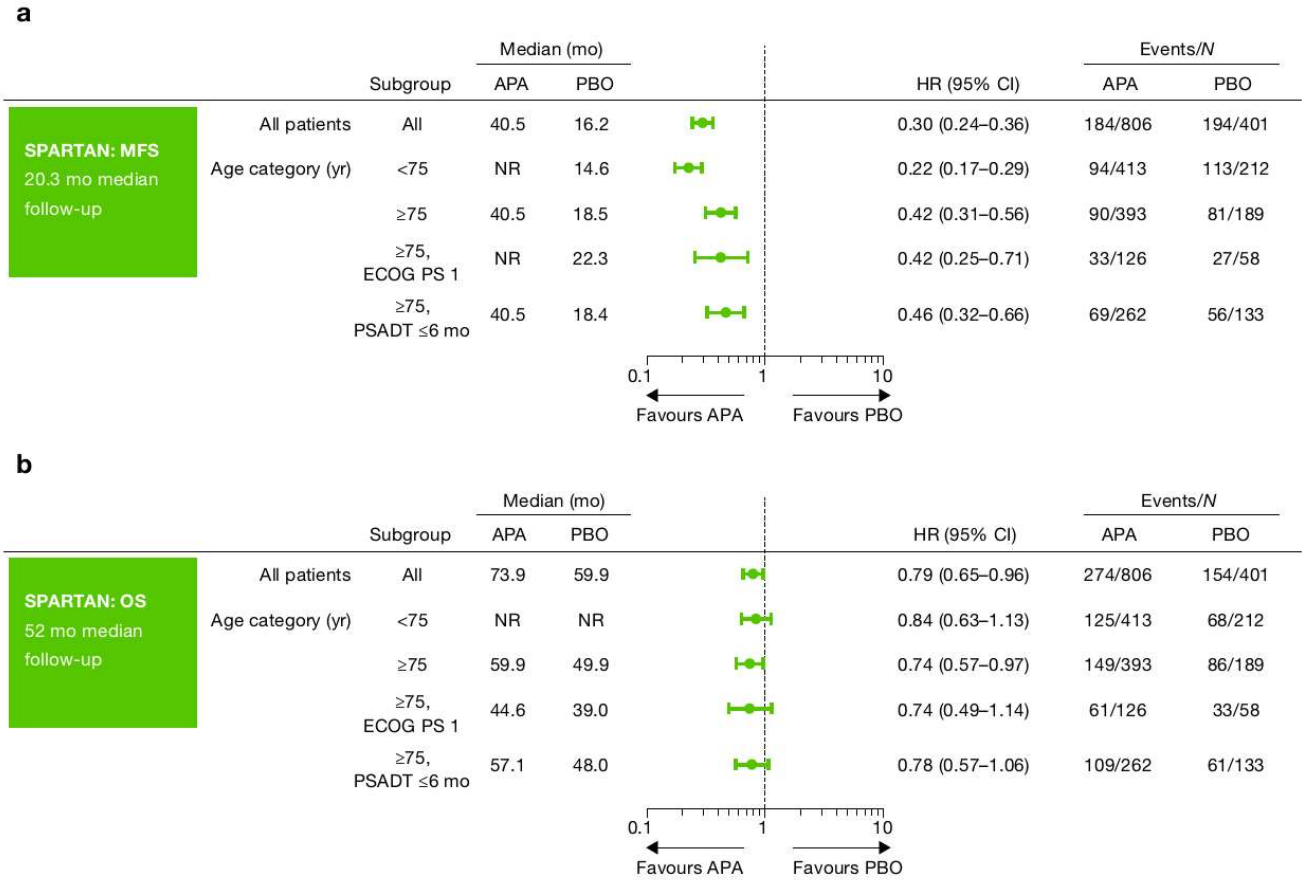

Fig. S5 LS mean change from baseline in patient-reported physical wellbeing and energy levels in TITAN and SPARTAN by <65, 65–79 and ≥80 year age groups. **(a)** FACT-P Physical Wellbeing, consisting of questions GP1 “I have a lack of energy”, GP2 “I have nausea”, GP3 “Because of my physical condition, I have trouble meeting the needs of my family”, GP4 “I have pain”, GP5 “I am bothered by the side effects of treatment”, GP6 “I feel ill” and GP7 “I am forced to spend time in bed”. **(b)** FACT-P question GP1 “I have a lack of energy”. Bars show standard error, dotted horizontal lines show clinically meaningful change from baseline at visits that had >10% of patients completing FACT-P. APA apalutamide, FACT-P Functional Assessment of Cancer Therapy-Prostate, LS least squares, PBO placebo, PRO patient-reported outcome.

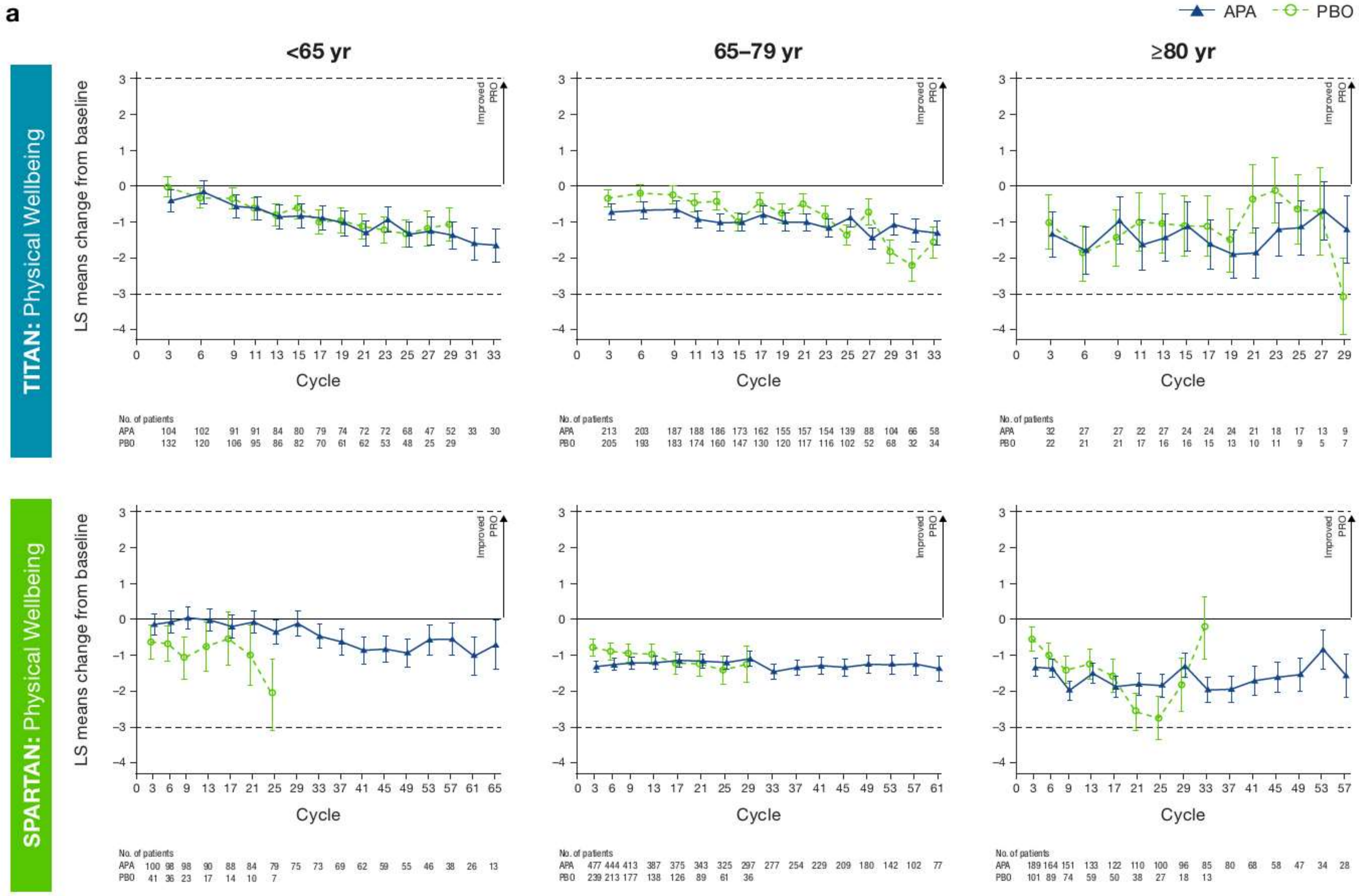
a
APA
PBO
<65 yr
65–79 yr
≥80 yr
TITAN: Physical Wellbeing
SPARTAN: Physical Wellbeing
LS means change from baseline
Improved PRO
Cycle
No. of patients
APA 104 102 91 91 84 80 79 74 72 72 68 47 52 33 30
PBO 132 120 106 95 86 82 70 61 62 53 48 25 29
No. of patients
APA 213 203 187 188 186 173 162 155 157 154 139 88 104 66 58
PBO 205 193 183 174 160 147 130 120 117 116 102 52 68 32 34
No. of patients
APA 32 27 27 22 27 24 24 24 21 18 17 13 9
PBO 22 21 21 17 16 16 15 13 10 11 9 5 7
No. of patients
APA 100 98 98 90 88 84 79 75 73 69 62 59 55 46 38 26 13
PBO 41 36 23 17 14 10 7
No. of patients
APA 477 444 413 387 375 343 325 297 277 254 229 209 180 142 102 77
PBO 239 213 177 138 126 89 61 36
No. of patients
APA 189 164 151 133 122 110 100 96 85 80 68 58 47 34 28
PBO 101 89 74 59 50 38 27 18 13

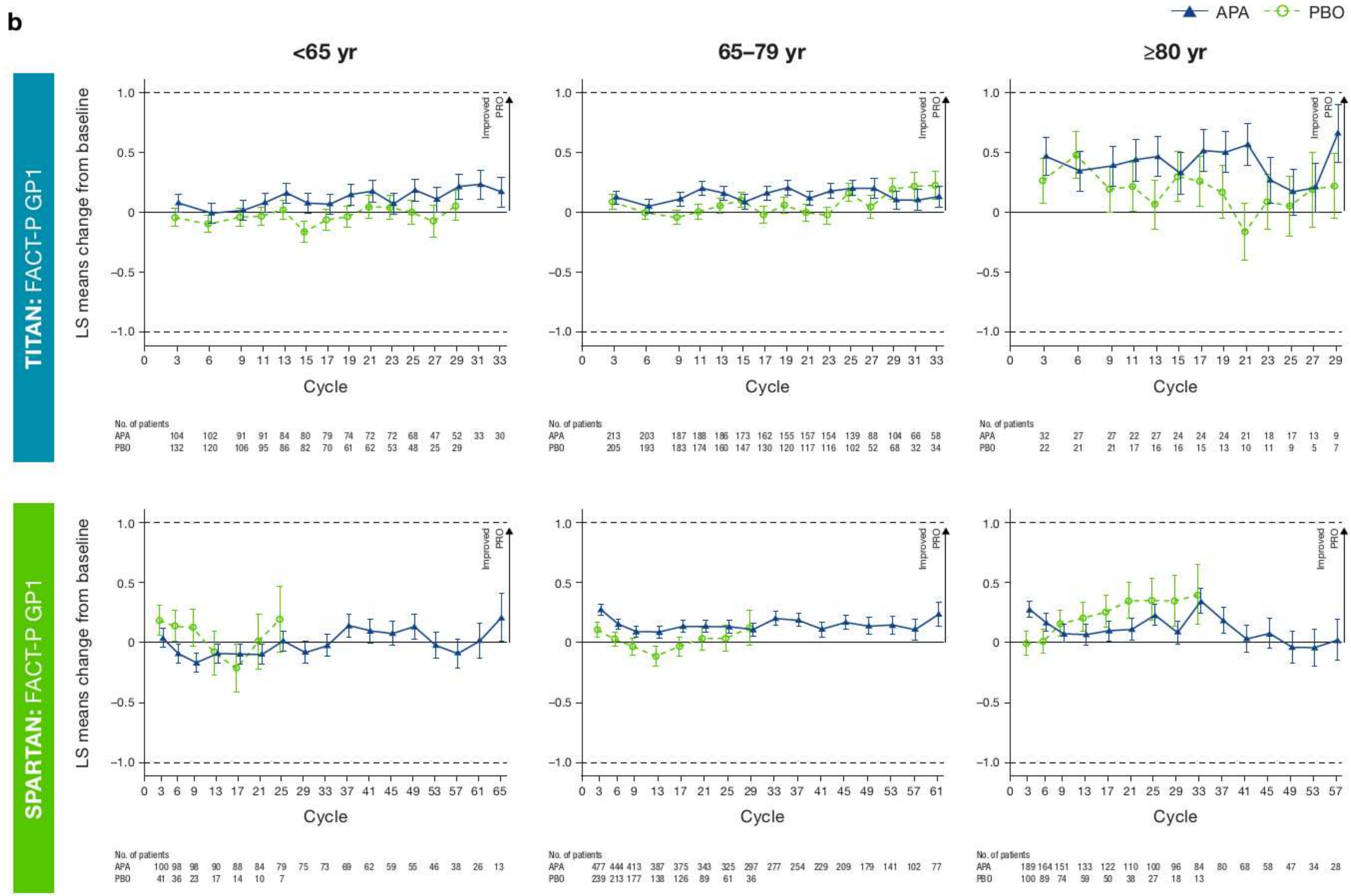
b
APA
PBO
<65 yr
65–79 yr
≥80 yr
TITAN: FACT-P GP1
SPARTAN: FACT-P GP1
LS means change from baseline
Improved PRO
Cycle
No. of patients
APA 104 102 91 91 84 80 79 74 72 72 68 47 52 33 30
PBO 132 120 106 95 86 82 70 61 62 53 48 25 29
No. of patients
APA 213 203 187 188 186 173 162 155 157 154 139 88 104 66 58
PBO 205 193 183 174 160 147 130 120 117 116 102 52 68 32 34
No. of patients
APA 32 27 27 22 27 24 24 24 21 18 17 13 9
PBO 22 21 21 17 16 16 15 13 10 11 9 5 7
No. of patients
APA 100 98 98 90 88 84 79 75 73 69 62 59 55 46 38 26 13
PBO 41 36 23 17 14 10 7
No. of patients
APA 477 444 413 387 375 343 325 297 277 254 229 209 179 141 102 77
PBO 239 213 177 138 126 89 61 36
No. of patients
APA 189 164 151 133 122 110 100 96 84 80 68 58 47 34 28
PBO 100 89 74 59 50 38 27 18 13

Fig. S6 Least squares mean change from baseline in patient-reported FACT-P total score in TITAN and SPARTAN by age <75 years versus ≥75 years. Bars show standard error, dotted horizontal lines show clinically meaningful change from baseline. APA apalutamide, FACT-P Functional Assessment of Cancer Therapy-Prostate, LS least squares, PBO placebo, PRO patient-reported outcome.

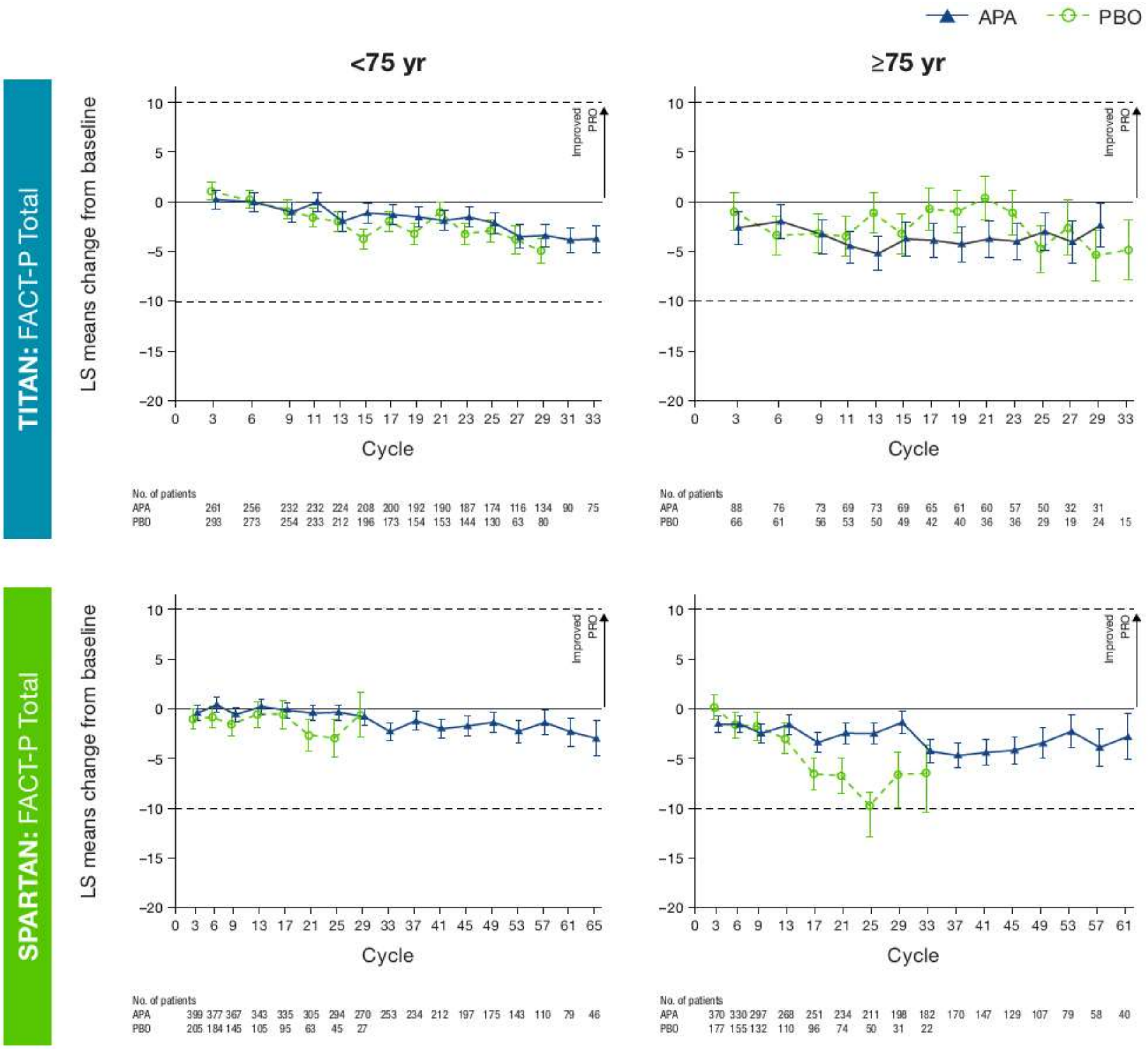

Supplement: Supplementary file 1 — Shen TITAN/SPARTAN Older Pts_Supplement [file 41416_2023_2492_MOESM1_ESM.pdf]
